# Asymptomatic malaria infection among pregnant women attending antenatal care in malaria endemic areas of North-Shoa, Ethiopia: a cross-sectional study

**DOI:** 10.1186/s12936-020-3152-9

**Published:** 2020-02-11

**Authors:** Daniel Getacher Feleke, Aderaw Adamu, Angesom Gebreweld, Melkam Tesfaye, Wondmagegn Demisiss, Genet Molla

**Affiliations:** Departmentof Medical Laboratory Science, College of Medicine and Health Sciences, Wollo University, Dessie, Ethiopia

**Keywords:** Asymptomatic malaria, Anaemia, Pregnant women, Prevalence, Plasmodium species, Ethiopia

## Abstract

**Background:**

The effort to reduce the burden of malaria should target transmission in the community by accurate identification of asymptomatic infections. In malaria-endemic areas, asymptomatic malaria infection is still associated with complications. Malaria during pregnancy is characterized by anaemia and placental malaria, leading to low birth weight and perinatal morbidity and mortality. This study aimed to provide reliable data on the burden of asymptomatic malaria among pregnant women in malaria endemic areas of North-Shoa, Ethiopia.

**Methods:**

Cross-sectional study was carried out to assess the prevalence and predictors of asymptomatic malaria in pregnant women from November 2018 to January 2019. Multistage sampling technique was employed to include 263 study participants. Data were analysed using SPSS version 20.0 statistical software. In all comparisons, p-values ≤ 0.05 was considered as statistically significant.

**Results:**

The prevalence of asymptomatic malaria infection was 5.7% (15/263) and 3.4% (9/263) by using microscopy and RDTs, respectively. *Plasmodium falciparum* was a dominant species 9 (3.4%) and *Plasmodium vivax* accounted for 6 (2.3%) of *Plasmodium* infections as detected by microscopy. Multivariate analysis showed that ITN usage and haemoglobin level had a statistically significant association with *Plasmodium* infection after adjusting other possible factors. Compared to those who were using ITN always, the odds of *Plasmodium* infection was 18.16 times higher (95% CI 1.84–179.07) in pregnant women who were not using ITN, and 5.19 times higher (95% CI 0.55–49.21) in pregnant women who were using ITN sometimes. Asymptomatic malaria infected pregnant women were 3.78 times (95% CI 0.98–14.58) more likely to be anaemic compared to non-infected pregnant women.

**Conclusion:**

The present study showed asymptomatic malaria is prevalent in pregnant women and it has statistically significance association with the haemoglobin level of pregnant women. This indicates pregnant women have to be screened for asymptomatic malaria to avoid health consequences of malaria infection during pregnancy for the mother and fetus.

## Background

There is an estimated 219 million malaria cases globally and 435,000 deaths due to malaria each year with the highest mortality reported in Africa [1]. Malaria infection during pregnancy is a major public health problem in tropical and subtropical regions [2]. It affects an estimated 24 million pregnant women in sub-Saharan Africa annually [3]. In malaria-endemic areas, malaria is the cause for almost 25% of maternal deaths each year, with the greatest risk of infection and morbidity occurring in primiparous women, adolescents, and those co-infected with human immunodeficiency virus (HIV) [4]. Malaria during pregnancy may cause a variety of adverse consequences including maternal anaemia, placental accumulation of parasites, low birth weight from prematurity and intrauterine growth retardation, congenital infection and infant mortality [5]. Asymptomatic infection with *Plasmodium* species is common in malaria endemic areas [6, 7]. The prevalence of asymptomatic parasitaemia reaches over 90% in children, in highly endemic areas of Africa [8]. Asymptomatic individuals, whether with a detectable parasitaemia by microscopy or below the microscopic detection level, can be a reservoir for transmission by *Anopheles* mosquitoes and may progress to symptomatic disease [8, 9]. Moreover, untreated asymptomatic malaria evolves in a chronic infection characterized by marked dyserythropoietic changes in the red cell precursors and increased erythrophagocytosis [10]. The efforts to reduce the burden of malaria require moving beyond the treatment of clinical infections to targeting transmission in the community by accurate identification of asymptomatic infections [11].

Approximately, 75% of the landmass of Ethiopia is estimated to be “malarious” with about 52 million people living in these areas and about four to five million people affected by malaria annually. *Plasmodium falciparum* and *Plasmodium vivax* account for 60–70% and 30–40% of cases, respectively [12–14]. In Ethiopia, early diagnosis and prompt treatment is considered as key strategy for malaria control. Currently, special attention is given to the reduction of maternal mortality and reduction of malaria cases. Therefore, this study aimed to provide reliable data on the burden of asymptomatic malaria among pregnant women in malaria endemic areas of North-Shoa, Ethiopia.

## Methods

### Study area

North-Shoa is one of the 10 Zones in the Ethiopian Amhara Regional state. Based on the 2007 Census conducted by the Central Statistical Agency of Ethiopia (CSA), this Zone has a total population of 1,837,490, of whom 928,694 are men and 908,796 women. The zone has an area of 15,936.13 square kilometers. Thirty-nine percent (39%) of the zone is exposed to malaria. Among the malaria endemic areas of the zone Karakore, Ataye and Senbete towns were selected. These towns are among the hot areas in North-Shoa zone and commonly affected by malaria. The towns have governmental and non-governmental health facilities. Considering the governmental health facilities, Ataye town has one district hospital and one health centre, while Karakore and Senbete town has one health centre each. These health institutions give service for the patients in the towns and their surrounding areas.

### Study design and study subjects

This cross-sectional study was conducted in malaria endemic areas of North-Shoa, Ethiopia from November 2018 to January 2019. The study was conducted among apparently healthy pregnant women. Pregnant women with absence of disease symptom/sign within the past 48 h, axillary temperature ≤ 37.5 °C, permanent residents in the area, and those willing to participate in the study and signed the informed consent were included. Individuals treated with anti-malarial drugs in the previous 6 weeks prior to data collection and those who undergo any kind of long-term medical treatments, and unwilling individuals were excluded.

### Sample size determination and sampling technique

Sample size was calculated using asymptomatic malaria prevalence of 9.1% reported from a study conducted in the rural surroundings of Arbaminch Town [6]. The random error of 5% and confidence level of 95% were assumed in the sample size determination. The calculated sample size was 127. By taking a design effect of 2, the total sample size became 254. Nine additional pregnant women were recruited to enhance the reliability of the study.

**Fig. 1:**
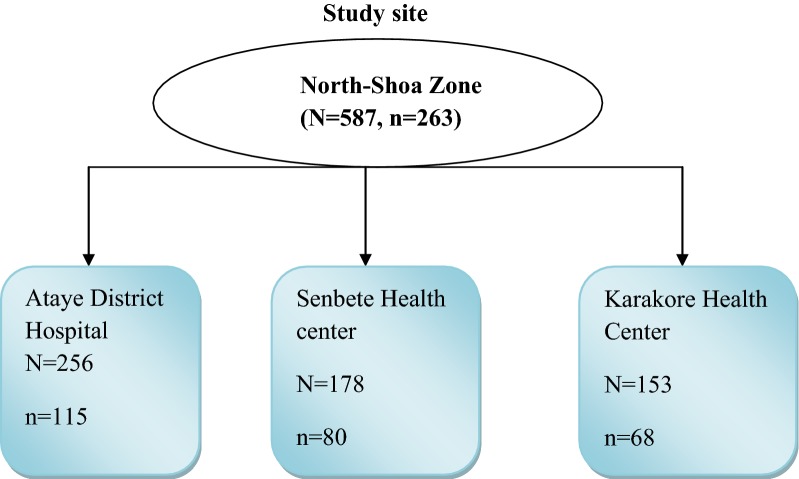
Sampling procedure for a study conducted on asymptomatic malaria among pregnant women in malaria endemic areas of North-Shoa, North-East Ethiopia from November 2018 to January 2019

Multistage sampling technique was used to select study subjects. Three malaria endemic areas were selected by simple random sampling using the lottery method from the known five malarious areas of North-Shoa zone. From each selected areas one health institution was selected by simple random sampling using lottery method. The three selected health institutions were Ataye district hospital, Senbete Health Center and Karakore Health Centres. The sample size was then distributed proportionally to the selected Health Centres based on the size of their pregnant women who are following antenatal care. In the selected health centre, the pregnant women were selected by simple random sampling by using the sampling frame (Fig. 1).

### Data collection

Data were collected using microscopy and RDT methods to diagnose asymptomatic malaria in pregnant women. Structured questionnaire was used to collect socio-demographic and other associated factors. To ensure reliable data collection the questionnaire was pre-tested before the actual data collection and training was given to data and sample collectors on data/sample collection procedures.

### Laboratory investigation

Thin and thick blood smears were prepared, stained with 3% Giemsa and examined microscopically from each asymptomatic pregnant woman. Thin smears were considered positive for malaria if one or more malarial parasites were seen; and, negative if no asexual form of *Plasmodium* was observed in 200 high-power fields. On the other hand, thick blood films were taken as positive if one or more malaria parasites have been observed; and, negative if no parasites were seen after examining 1000 white blood cells.

Malaria diagnosis using CareStart Combo malaria (Access BIO, Inc. Monmouth junction New Jersey 08852) test was performed. Rapid diagnostic test results were read by the laboratory technicians. The result was read by a second experienced laboratory technologist who was unaware of the result. In case of disagreement it was read by the principal investigators. Positive and negative blood samples were collected and RDTs were stored in appropriate temperature and humidity and it was checked throughout the study for its validity.

### Data analysis

Data were entered and analysed using SPSS version 20 statistical package. Frequencies were used to determine the prevalence of asymptomatic malaria in pregnant women. Binary logistic regression was performed to assess the predictors of asymptomatic *Plasmodium* infection. In the univariate analysis variables significant at *P* value of 0.25 were entered to multivariate logistic regression analysis model. P-value less than 0.05 were considered statistically significant in all comparisons.

## Results

### Socio-demographic characteristics of pregnant women

A total of 263 pregnant women were involved in this study. The age of the study participants were ranged from 16 to 41 years with the mean age of 27.8 years (SD = ± 5.34). Majority of study participants were house wives (39.2%) and 95.4% were married. The mean family size in the present study was 3.28 (SD ± 1.35). About 36.5% the pregnant women were multigravidae, 52.5% of them were in their second trimester of pregnancy and majority (96.6%) of them were following antenatal care (Table 1).

**Table 1:** Scio-demographic characteristics of pregnant women in malaria endemic areas of North-Shoa, Ethiopia

Variables	Number (%)	Variables	Number (%)
*Age groups*		*Marital status*	
< 20	17 (6.5)	Single	5 (1.9)
≥ 20	246 (93.5)	Married	251 (95.4)
*Occupation*		Divorced	7 (2.7)
Farmer	69 (26.2)	*Parity*	
Merchant	62 (23.6)	Primigravidae	78 (29.7)
House wife	103 (39.2)	Secundgravidae	89 (33.8)
Government employee	29 (11.0)	Multigravidae	96 (36.5)
*Educational status*		*Gestational age*	
Illiterate	49 (18.6)	1st trimester	16 (6.1)
Read/write	52 (19.8)	2nd trimester	138 (52.5)
Elementary	76 (28.9)	3rd trimester	109 (41.4)
Secondary	57 (21.7)	*ANC follow up*	
College and above	29 (11.0)	Yes	254 (96.6)
*Residence*		No	9 (3.4)
Urban	106 (40.3)	*Family size*	
Rural	157 (59.7)	1–3	153 (58.2)
*ITN use*		≥ 4	110 (41.8)
Always	89 (33.8)	*Insecticide spray (past 12* *months)*	
Sometimes	112 (42.6)	Yes	142 (54)
Never	62 (23.6)	No	121 (46)

### Asymptomatic malaria infection among pregnant women

The prevalence of asymptomatic malaria infection was 5.7% (15/263) and 3.4% (9/263) by microscopy and RDTs, respectively. *Plasmodium falciparum* was a dominant species with 9/15 and *P. vivax* accounted for 6/15 of *Plasmodium* infection as detected by microscopy. Mixed infection was not detected by both microscopy and RDTs. In this study, there was a good measure of agreement between microscopy and CareStart Combo malaria (Access BIO, Inc. Monmouth junction New Jersey 08852) test with a specificity and sensitivity above 90%. Forty-six percent (46%) of pregnant women had a haemoglobin level of < 11 g/dL and 13.0% of them were infected with malaria.

Socio-demographic characteristics and other predictors were analyzed using binary logistic regression. In univariate analysis, family size, ITN usage and haemoglobin level showed significant association with *Plasmodium* infection at P-value of < 0.05 (Table 2). In multivariate analysis only ITN usage and haemoglobin level had a statistically significant association with *Plasmodium* infection among pregnant women after adjusting other possible factors. Compared to women who always used ITNs, the odds of infection were 18.16 times higher among pregnant women who never used ITNs during their current pregnancy (95% CI 1.84–179.07). Among women who used ITNs sometimes the odds of infections were 5.19 times higher (95% CI 0.55–49.21) when compared to women who always used ITNs. There was statistically significant association between anaemia and asymptomatic malaria infection both in univariate and multivariate analysis. Asymptomatic malaria infected pregnant women were almost 4 times (95% CI 1.98–14.58) more likely to be anaemic compared to non-infected pregnant women.

**Table 2:** Multivariate logistic regression analysis of predictors for asymptomatic Plasmodium infection among pregnant woman in malaria endemic areas of North-Shoa, North-East, Ethiopia

Variables	No. examined (%)	Pos (%)	COR	P-value	AOR	P-value
*Occupation*						
Farmer	69 (26.2)	5 (9.1)	1.72 (0.18–16.12)	0.63	0.25 (0.01–10.96)	0.47
Merchant	62 (23.6)	6 (9.7)	3.56 (0.42–30.41)	0.24	1.13 (0.05–24.86)	0.93
House wife	103 (39.2)	3 (2.9)	0.84 (0.08–8.39)	0.88	0.28 (0.01–7.98)	0.45
Government employee	29 (11.0)	1 (3.4)	1		1	
*Educational status*						
Illiterate	49 (18.6)	4 (8.2)	2.49 (0.27–23.41)	0.43	3.96 (0.10–159.03)	0.46
Read/write	52 (19.8)	1 (1.9)	0.55 (0.03–9.12)	0.68	1.25 (0.03–62.14)	0.91
Elementary	76 (28.9)	3 (3.9)	1.15 (0.12–11.53)	0.91	0.68 (0.03–17.61)	0.81
Secondary	57 (21.7)	6 (10.5)	3.29 (0.38–28.75)	0.28	6.55 (0.30–143.56)	0.23
College and above	29 (11.0)	1 (3.4)	1		1	
*Parity*						
Primigravidae	78 (29.7)	3 (3.8)	0.29 (0.06–1.41)	0.12	1.32 (0.11–16.28)	0.82
Secundgravidae	89 (33.8)	5 (5.6)	0.66 (0.21–2.08)	0.47	1.93 (0.33–11.46)	0.47
Multigravidae	96 (36.5)	7 (7.3)	1		1	
*Family size*						
1–3	153 (58.2)	4 (2.6)	1		1	
≥ 4	110 (41.8)	11 (10.0)	4.14 (1.28–13.37)	0.02*	4.94 (0.67–36.41)	0.11
*Haemoglobin*						
Anaemic (< 11 g/dL)	46 (17.5)	6 (13.0)	3.47 (1.17–10.28)	0.02*	3.78 (1.98–14.58)	0.045*
Not anaemic (≥ 11 g/dL)	217 (82.5)	9 (4.1)	1		1	
*ITN usage*						
Always	89 (33.8)	1 (1.1)	1		1	
Sometimes	112 (42.6)	5 (4.5)	4.11 (0.47–35.85)	0.20	5.19 (0.55–49.21)	0.15
Never	62 (23.6)	9 (14.5)	14.94 (1.84–121.28)	0.01*	18.16 (1.84–179.07)	0.01*
*Insecticide spray (past 12* *months)*						
Yes	142 (54)	4 (2.8)	1		1	
No	121 (46)	11 (9.1)	2.47 (0.82–7.43)	0.11	3.04 (0.88–10.56)	0.12

Although there was no statistically significant association between malaria infection and indoor spraying in the past 12 months, pregnant women who did not use indoor spray were 3.04 times (95% CI 0.88–10.56) more likely to be infected by *Plasmodium* (Table 2).

## Discussion

In the present study, the prevalence of asymptomatic *Plasmodium* infection among pregnant women was 5.7% and 3.4%by using Giemsa-stained blood smear microscopy and CareStart™ HRP2/pLDH combo rapid malaria test, respectively. This prevalence was lower than the finding reported from the rural surroundings of Arbaminch town, South Ethiopia, which was 9.1% and 9.7% by microscopy and RDT, respectively among pregnant women [6]. This might be because the area of the present study is among the high malaria transmission areas in Ethiopia. In areas with high malaria transmission, individuals frequently exposed to malaria infection which leads to development of protective immunity that makes plasmodium infection to be asymptomatic [6]. While, individuals living in lower transmission areas are not frequently exposed to malaria infection frequently and when exposed, they develop symptomatic malaria. In addition, the difference in the sample size, sampling technique, and the study setting such as geographical differences, altitude and temperature could bring the difference in asymptomatic malaria prevalence. Another main reason for the lower asymptomatic malaria prevalence in this study might be due to the data collection period which was during minor malaria transmission period in the study area.

On the other hand, the prevalence of asymptomatic malaria in the present study was higher than the prevalence of 3.1% by microscopy and 4.8% by RDTs reported from Nigeria teaching hospital [15]. The difference of study setting such as geographical differences, altitude and temperature could partially explain the difference in asymptomatic malaria prevalence.

The prevalence of asymptomatic *Plasmodium* infection was higher using microscopy than RDT which disagrees with a report from the rural surroundings of Arbaminch Town, South Ethiopia [6]. However, the finding was consistent with the study findings reported from Tanzania and Myanmar [16, 17].

There was statistically significant association between ITN usage habit and plasmodium infection in pregnant women. This was in agreement with the finding reported from the rural surroundings of Arbaminch town [6]. The odds of *Plasmodium* infection were higher in pregnant women who have no habit of ITN usage than those who used nets always. This finding is in line with the fact that effectiveness of insecticide-treated net usage is considered as one of the most important prevention and control tools [18]. In contrast to the findings in this study, the study conducted in Burkina Faso reported no statistically significant association between asymptomatic malaria infection and insecticide-treated nets usage habit [19].

## Conclusion

The present study showed asymptomatic malaria is prevalent in pregnant women and it has statistically significance association with the haemoglobin level of pregnant women. This indicates pregnant women have to be screened for asymptomatic malaria to avoid health consequences of malaria infection during pregnancy for the mother and fetus.

## Data Availability

The authors confirm that all data underlying the findings are fully available without restriction. All relevant data are within the manuscript.
